# Intensified MR-guided pulsed adaptive cancer treatment- a potential new treatment option for large(>5cm) central non-small cell lung cancer, a case report

**DOI:** 10.3389/fonc.2026.1791499

**Published:** 2026-06-15

**Authors:** Maciej Blok, Tomasz Wisniewski, Izabela Wiatrowska, Maciej Harat

**Affiliations:** 1 Department of Neurooncology and Radiosurgery, Franciszek Lukaszczyk Memorial Oncology Center, Bydgoszcz, Poland; 2 Department of Clinical Medicine, Jan and Jedrzej Sniadecki University of Science and Technology, Bydgoszcz, Poland; 3 Department of Oncology and Brachytherapy, Nicolaus Copernicus University, Ludwik Rydygier Collegium Medicum, Bydgoszcz, Poland; 4 Department of Medical Physics, Franciszek Lukaszczyk Memorial Oncology Center, Bydgoszcz, Poland

**Keywords:** adaptation, LINAC, lung, radiotherapy, SBRT

## Abstract

**Background:**

There are currently no effective treatment options for large, inoperable, centrally located thoracic lesions with poor or suboptimal response to immunotherapy. The use of SBRT for tumors larger than 5 cm in this location is limited by toxicity. Therefore, new and innovative treatment strategies are a major unmet medical need. Adaptive radiotherapy (ART) guided by MR-Linac systems, enables real-time adjustment of the treatment plan according to daily anatomical changes and tumor response. This approach is particularly beneficial in thoracic oncology, where tumor shrinkage and respiratory motion can significantly affect dose delivery. Moreover, ART may enhance the therapeutic synergy between radiotherapy and immunotherapy.

**Case presentation:**

We report the case of a 65-year-old woman diagnosed with stage IV non-small cell lung cancer (NSCLC) of mixed squamous and adenocarcinoma histology. The patient initially presented with seizures, and an MRI revealed 10 intracranial metastases alongside a 55 mm tumor in the left lung. Following stereotactic brain radiotherapy and the initiation of nivolumab plus ipilimumab, the treatment was continued with nivolumab monotherapy due to grade 3 pulmonary toxicity. Imaging demonstrated a tumor progression limited to the lung. She was then qualified for MR-guided adaptive stereotactic radiotherapy (3 × 10 Gy), delivered at 10-day intervals. Significant tumor regression required redefinition of the GTV before fractions 2 and 3. No acute or late toxicity was observed. A 6-month follow-up PET-CT revealed marked regression in tumor size (from 79 mm to 17 mm) and metabolic activity (SUVmax reduced from 33.1 to 5.13). Comparative imaging showed less extensive post-radiation lung changes than typically observed after conventional radiotherapy.

**Conclusion:**

This case illustrates the potential of MR-guided adaptive radiotherapy to enhance treatment precision, reduce toxicity, and enable immune-radiotherapy synergy. Integration of the PULSAR concept—with spaced fractions and immunotherapy—may improve tumor response. Further clinical trials are required to confirm these benefits on larger patient populations.

## Introduction

There are currently no effective treatment options for large, inoperable, centrally located thoracic lesions with poor or suboptimal response to immunotherapy. The use of SBRT for tumors larger than 5 cm in this location is limited by toxicity. Adaptive radiotherapy (ART) is an advanced technique that enables real-time modification of treatment plans based on daily anatomical changes, tumor shrinkage, and organ motion. By accounting for both inter- and intrafraction variability, ART improves target coverage while reducing the dose to surrounding healthy tissues and critical structures (1). This approach is particularly valuable in the management of large tumors or those located in anatomically complex regions, where conventional radiotherapy often requires broader margins and carries a higher risk of complications. Magnetic resonance–guided radiotherapy (MRgRT), delivered via integrated MR-Linac systems, provides high-resolution imaging, precise tumor tracking, and on-table adaptive planning. Furthermore, combining stereotactic body radiotherapy (SBRT) with immunotherapy has shown promise in enhancing treatment response. Radiotherapy may augment systemic immune activity by increasing tumor antigen release and modulating the tumor microenvironment (2). This case report describes a novel strategy for the management of an inoperable, large, oligometastatic stage IV non-small cell lung cancer (NSCLC), with a particular focus on treatment adaptation, integration of immunotherapy, and toxicity outcomes.

## Case presentation

### Patient characteristics

A 65-year-old woman was initially diagnosed in April 2024 following a seizure. Brain MRI revealed 10 intracranial metastatic lesions and a 55 mm tumor in the left lung. Histopathological examination confirmed non-small cell lung cancer (NSCLC) of mixed histology—both squamous and adenocarcinoma components. Stereotactic radiotherapy was administered to the metastatic brain lesions. A follow-up brain MRI demonstrated a partial regression of the intracranial metastases. CT scans of the chest, abdomen, and pelvis showed no evidence of additional metastatic disease. The patient was started on a combination of immunotherapy with nivolumab and ipilimumab. Due to the development of grade 3 pulmonary toxicity, treatment was continued with nivolumab monotherapy. A subsequent PET-CT revealed tumor progression in the left lung, without evidence of new metastatic lesions elsewhere. Following a multidisciplinary tumor board review, the patient was qualified for adaptive radiotherapy using MR-Linac. At the time of qualification, her general condition was good, with a Karnofsky Performance Status (KPS) of 90. She recently reported a worsening of exercise tolerance but had no neurological symptoms.

### Treatment planning and delivery

PET-CT, chest MRI, and non-contrast chest CT were used for radiotherapy treatment planning. During radiotherapy preparation, a 4D-CT scan was performed and an internal target volume (ITV) was generated. As tumor motion was minimal (<2 mm in all directions), free-breathing CT acquisition was considered sufficient and was subsequently used for treatment planning. Due to the central location of the tumor, abdominal compression was not applied. Intrafractional tumor motion was continuously monitored during treatment using real-time MR imaging available on the MR-Linac system. Treatment was delivered using the 1.5 T Elekta Unity MR-Linac system with online adaptive replanning and real-time intrafraction MRI monitoring. The gross tumor volume (GTV) was delineated based on the anatomical extent of the tumor, with an initial GTV of 154.32 cm³ and a maximum tumor diameter of 79 mm. The planning target volume (PTV) was defined by expanding the GTV with a 5 mm isotropic margin, resulting in a PTV of 261.82 cm³. Dose constraints for organs at risk were adapted from established SBRT protocols for central thoracic lesions, primarily based on the experience reported by Timmerman et al. (3) and the RTOG 0813 recommendations (4). A total dose of 30 Gy was prescribed and delivered in three fractions. **Figure 1** illustrates the target volumes and corresponding doses to organs at risk (OARs).

**Figure 1:**
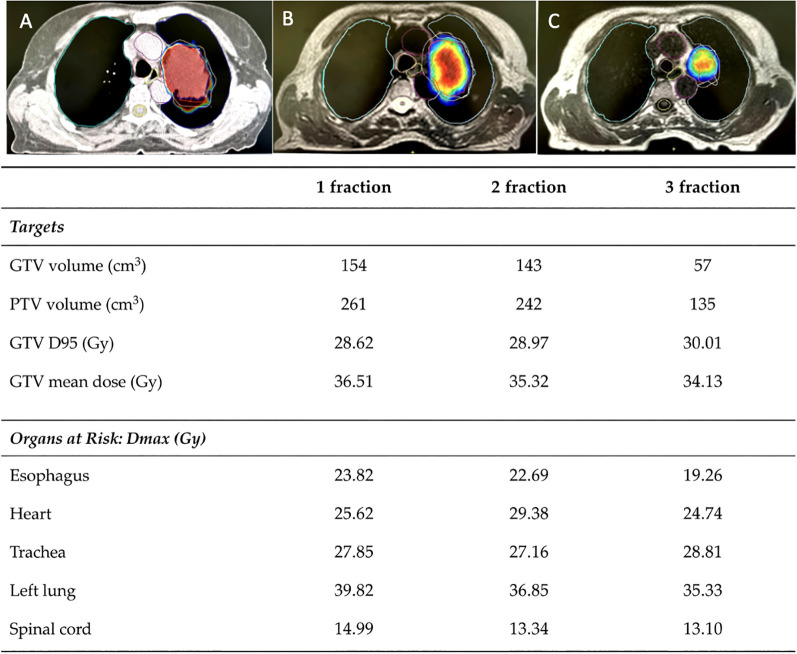
Imaging and dosimetric parameters for subsequent treatment fractions. **(A)** Axial CT image used for treatment planning of the first fraction. **(B, C)** Axial MRI images with dose distributions for the second and third fractions, respectively. The table summarizes target coverage parameters and maximum doses (Dmax) delivered to organs at risk for each consecutive fraction.

Fractions were administered at 10-day intervals. Nivolumab was continued as per the existing regimen at a dose of 360 mg and was administered three days after stereotactic body radiotherapy (SBRT) following the first and third fractions. The gross tumor volume (GTV) measured 154 cm³ at the first fraction, 143 cm³ at the second fraction, and 57 cm³ at the third fraction, reflecting a pronounced reduction in tumor size during treatment. Due to this significant regression, the GTV required redefinition before the second and third fractions. MRI was performed prior to each fraction and between fractions for treatment response assessment. No intrafractional re-planning was necessary, as the tumor motion during treatment was minimal. MRI images obtained during the adaptive planning and at the end of the treatment are presented in **Figure 2**.

**Figure 2:**
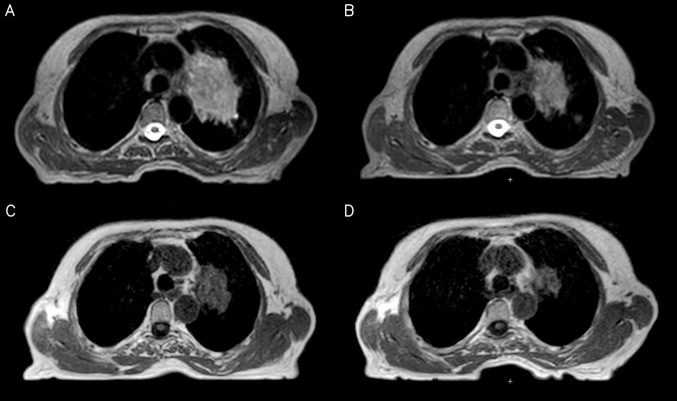
MRI images generated before adaptation on the MR LINAC system. **(A)** Tumor image before the 1st treatment fraction; **(B)** Tumor image before the 2nd treatment fraction **(C)** Tumor image before the 3rd treatment fraction; **(D)** Tumor image 10 days after the last radiotherapy fraction.

### Toxicity and tolerance

Early and late toxicity was assessed according to Common Terminology Criteria for Adverse Events (CTCAE v5.0). The patient reported no treatment-related symptoms immediately after radiotherapy, nor at the 3- and 6-month follow-up visits. At 3 months post-treatment, she reported a subjective improvement in dyspnoea.

### Follow-up and response assessment

A follow-up PET-CT, performed at 6-month follow-up, revealed marked both morphological and metabolic regression of the tumor. The maximum tumor diameter decreased from 79 mm to 17 mm, and SUVmax declined from 33.1 to 5.13. The findings are presented in **Figure 3**. The patient continues nivolumab monotherapy.

**Figure 3:**
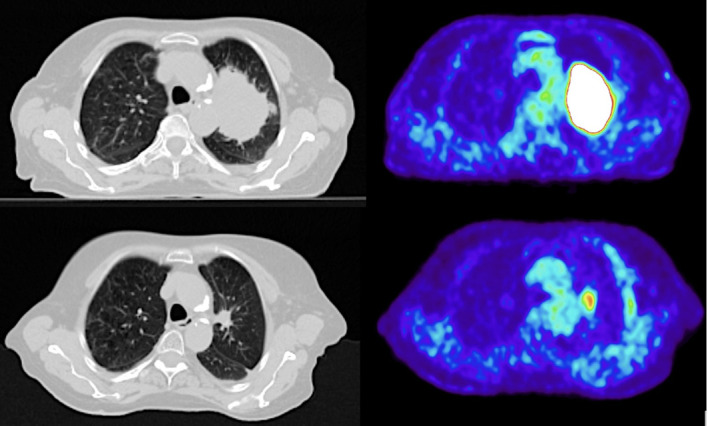
Treatment response. **(A)** CT scan for radiotherapy planning; **(B)** PET-CT scan for radiotherapy planning; **(C)** CT scan at 6 months post-treatment; **(D)** PET-CT scan at 6 months post-treatment. Notably, the surrounding lung parenchyma appears preserved, without the typical changes often observed after conventional radiotherapy.

## Discussion

This case illustrates the feasibility and safety of MR-guided adaptive radiotherapy (ART) in a patient with progressive stage IV NSCLC undergoing immunotherapy. Due to the large tumor volume and its central location, neither conventional fractionated radiotherapy nor standard SBRT dose schedules were considered feasible. The observed response—both morphological and metabolic—highlights the potential synergy between advanced radiation techniques and immune checkpoint blockade. In the present case, significant tumor regression necessitated redefinition of the gross tumor volume (GTV) before the second and third fractions, illustrating the benefit of adaptive planning in dynamically evolving tumor scenarios. The ability to modify the treatment plan without interrupting therapy improved both target coverage and sparing of adjacent organs at risk. Importantly, the treatment was well tolerated. No early or late radiotherapy-related toxicities were observed, and the patient reported improved respiratory capacity at follow-up. The selected prescription dose of 30 Gy delivered in 3 fractions was intentionally individualized for this patient due to the centrally located bulky tumor, close proximity to mediastinal critical organs, and concurrent nivolumab treatment. The aim was to maintain a biologically effective ablative dose while minimizing the risk of severe toxicity in this high-risk anatomical setting. This approach is consistent with previously published experiences regarding SBRT for central thoracic tumors and the PULSAR concept utilizing separated adaptive radiation pulses (3, 4). A comparison with other studies using Personalized Ultrafractionated Stereotactic Adaptive Radiotherapy demonstrates that different dose schedules and interfraction intervals have been applied. Eustace et al. described irradiation of a large left lung tumor with mediastinal lymph nodes to a total dose of 36 Gy delivered in 3 fractions with one-month intervals combined with concurrent pembrolizumab. No significant tumor shrinkage between irradiation pulses was observed in that report (5). In our study, a lower dose per fraction was used, while the interfraction interval was reduced to 10 days, consistent with the preclinical study using the murine Lewis Lung Carcinoma model (6). We observed a PTV reduction of 9% and 63% after the first and second fractions, respectively. This case also supports the theoretical benefit of the PULSAR approach, which combines MR-guided ultrafractionated radiotherapy with immunotherapy in spaced intervals. While this case provides valuable insight, it remains a single-patient report and lacks a long-term follow-up. Nevertheless, it adds to the growing body of evidence supporting MR-guided ART as a promising modality in the era of combined modality therapy. Large-scale clinical trials are needed to validate these findings and to establish optimal integration of MRgRT and immunotherapy in thoracic and other malignancies.

## Conclusion

This case illustrates the potential of MR-guided adaptive radiotherapy to enhance treatment precision, reduce toxicity, and enable immune-radiotherapy synergy. Integration of the PULSAR concept—with spaced fractions and immunotherapy—may improve tumor response. Further clinical trials are required to confirm these benefits on larger patient populations.

## Data Availability

The raw data supporting the conclusions of this article will be made available by the authors, without undue reservation.
